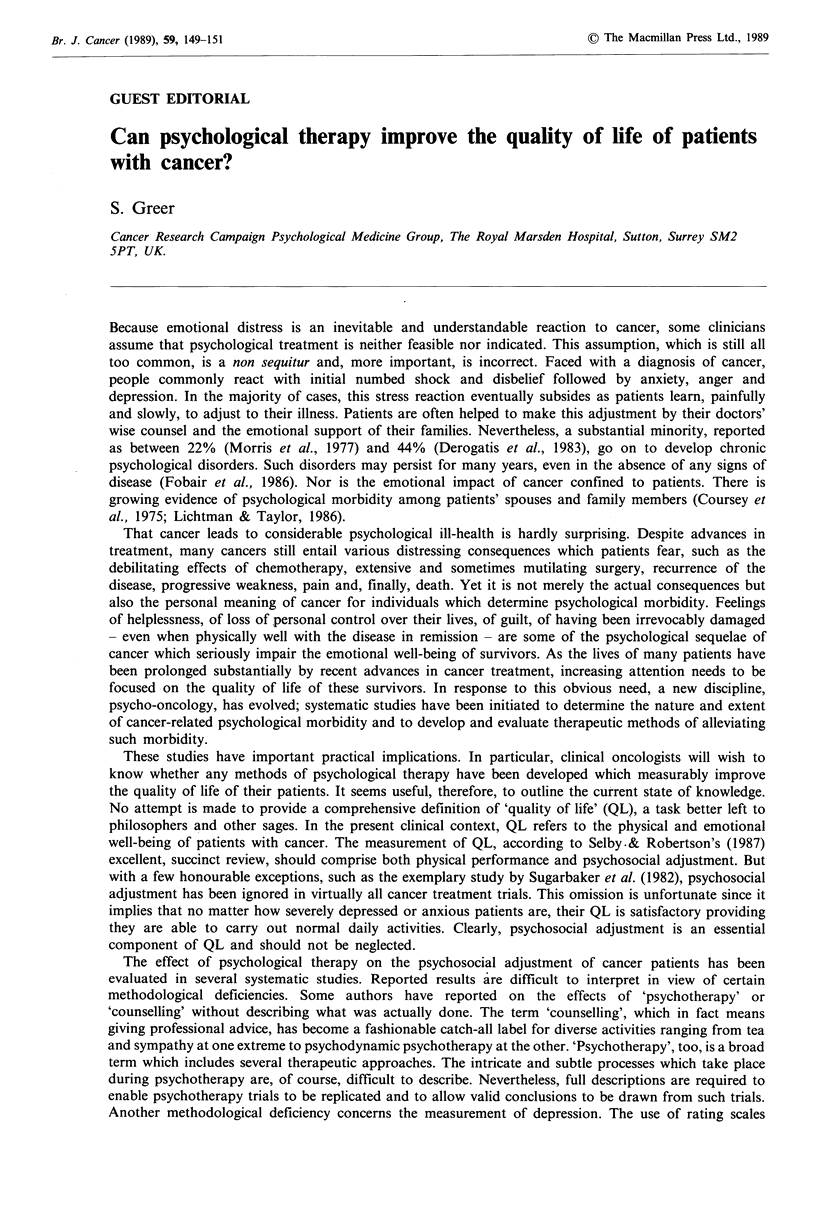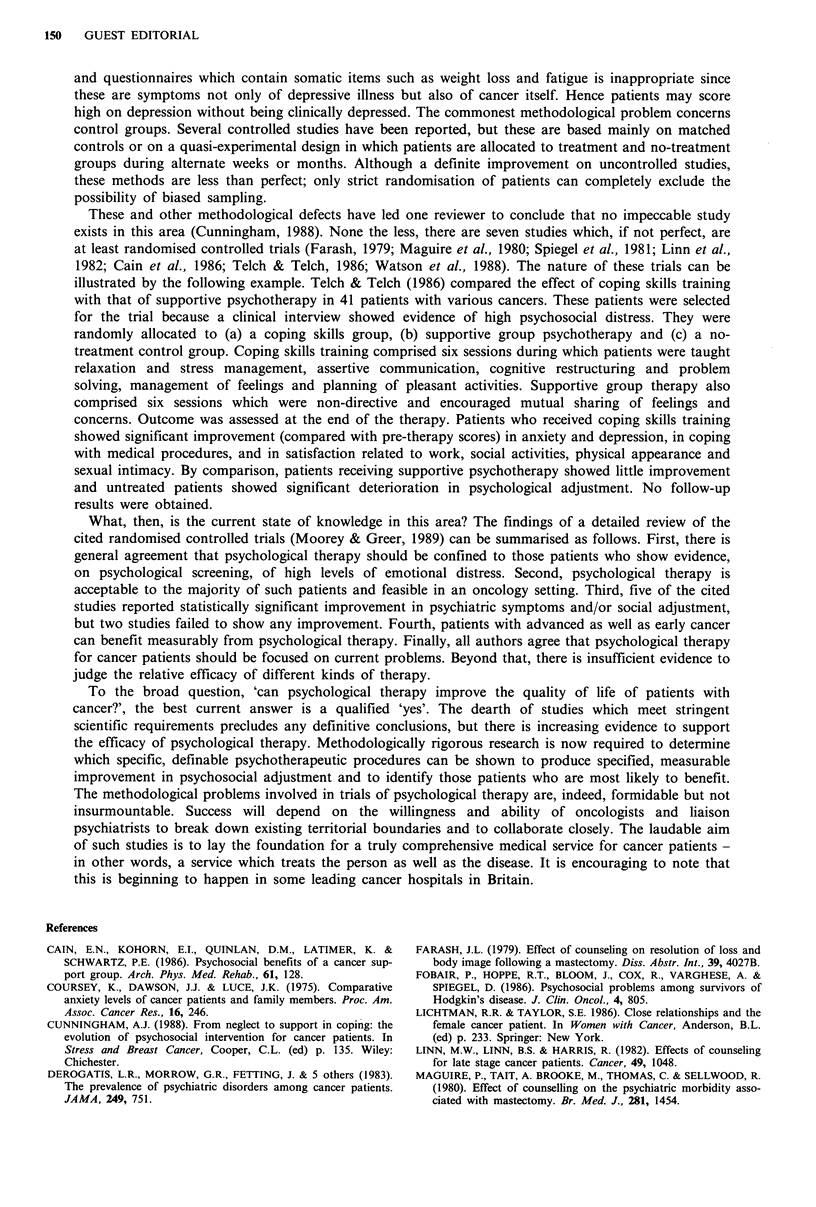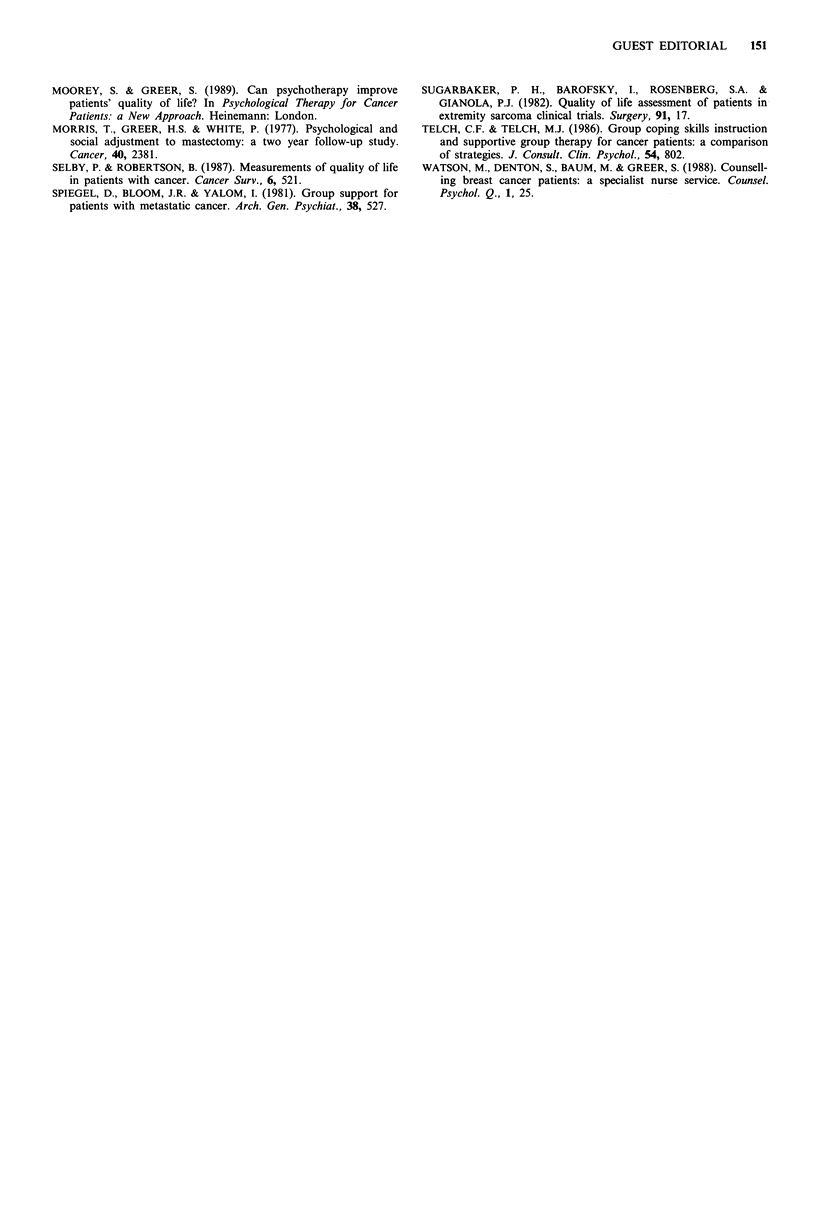# Can psychological therapy improve the quality of life of patients with cancer?

**DOI:** 10.1038/bjc.1989.31

**Published:** 1989-02

**Authors:** S. Greer

**Affiliations:** Cancer Research Campaign Psychological Medicine Group, Royal Marsden Hospital, Sutton, Surrey, UK.


Br. J. Cancer (1989), 59, 149–151 © The Macmillan Press Ltd., 1989

GUEST EDITORIAL

### Can psychological therapy improve the quality of life of patients with cancer?

S. Greer

*Cancer Research Campaign Psychological Medicine Group, The Royal Marsden Hospital, Sutton, Surrey SM2 5PT, UK.*

Because emotional distress is an inevitable and understandable reaction to cancer, some clinicians assume that psychological treatment is neither feasible nor indicated. This assumption, which is still all too common, is a *non sequitur* and, more important, is incorrect. Faced with a diagnosis of cancer, people commonly react with initial numbed shock and disbelief followed by anxiety, anger and depression. In the majority of cases, this stress reaction eventually subsides as patients learn, painfully and slowly, to adjust to their illness. Patients are often helped to make this adjustment by their doctors' wise counsel and the emotional support of their families. Nevertheless, a substantial minority, reported as between 22% (Morris *et al.*, 1977) and 44% (Derogatis *et al.*, 1983), go on to develop chronic psychological disorders. Such disorders may persist for many years, even in the absence of any signs of disease (Fobair *et al.*, 1986). Nor is the emotional impact of cancer confined to patients. There is growing evidence of psychological morbidity among patients' spouses and family members (Coursey *et al.*, 1975; Lichtman & Taylor, 1986).

That cancer leads to considerable psychological ill-health is hardly surprising. Despite advances in treatment, many cancers still entail various distressing consequences which patients fear, such as the debilitating effects of chemotherapy, extensive and sometimes mutilating surgery, recurrence of the disease, progressive weakness, pain and, finally, death. Yet it is not merely the actual consequences but also the personal meaning of cancer for individuals which determine psychological morbidity. Feelings of helplessness, of loss of personal control over their lives, of guilt, of having been irrevocably damaged – even when physically well with the disease in remission – are some of the psychological sequelae of cancer which seriously impair the emotional well-being of survivors. As the lives of many patients have been prolonged substantially by recent advances in cancer treatment, increasing attention needs to be focused on the quality of life of these survivors. In response to this obvious need, a new discipline, psycho-oncology, has evolved; systematic studies have been initiated to determine the nature and extent of cancer-related psychological morbidity and to develop and evaluate therapeutic methods of alleviating such morbidity.

These studies have important practical implications. In particular, clinical oncologists will wish to know whether any methods of psychological therapy have been developed which measurably improve the quality of life of their patients. It seems useful, therefore, to outline the current state of knowledge. No attempt is made to provide a comprehensive definition of 'quality of life' (QL), a task better left to philosophers and other sages. In the present clinical context, QL refers to the physical and emotional well-being of patients with cancer. The measurement of QL, according to Selby & Robertson's (1987) excellent, succinct review, should comprise both physical performance and psychosocial adjustment. But with a few honourable exceptions, such as the exemplary study by Sugarbaker *et al.* (1982), psychosocial adjustment has been ignored in virtually all cancer treatment trials. This omission is unfortunate since it implies that no matter how severely depressed or anxious patients are, their QL is satisfactory providing they are able to carry out normal daily activities. Clearly, psychosocial adjustment is an essential component of QL and should not be neglected.

The effect of psychological therapy on the psychosocial adjustment of cancer patients has been evaluated in several systematic studies. Reported results are difficult to interpret in view of certain methodological deficiencies. Some authors have reported on the effects of 'psychotherapy' or 'counselling' without describing what was actually done. The term 'counselling', which in fact means giving professional advice, has become a fashionable catch-all label for diverse activities ranging from tea and sympathy at one extreme to psychodynamic psychotherapy at the other. 'Psychotherapy', too, is a broad term which includes several therapeutic approaches. The intricate and subtle processes which take place during psychotherapy are, of course, difficult to describe. Nevertheless, full descriptions are required to enable psychotherapy trials to be replicated and to allow valid conclusions to be drawn from such trials. Another methodological deficiency concerns the measurement of depression. The use of rating scales and questionnaires which contain somatic items such as weight loss and fatigue is inappropriate since these are symptoms not only of depressive illness but also of cancer itself. Hence patients may score high on depression without being clinically depressed. The commonest methodological problem concerns control groups. Several controlled studies have been reported, but these are based mainly on matched controls or on a quasi-experimental design in which patients are allocated to treatment and no-treatment groups during alternate weeks or months. Although a definite improvement on uncontrolled studies, these methods are less than perfect; only strict randomisation of patients can completely exclude the possibility of biased sampling.

These and other methodological defects have led one reviewer to conclude that no impeccable study exists in this area (Cunningham, 1988). None the less, there are seven studies which, if not perfect, are at least randomised controlled trials (Farash, 1979; Maguire *et al.*, 1980; Spiegel *et al.*, 1981; Linn *et al.*, 1982; Cain *et al.*, 1986; Telch & Telch, 1986; Watson *et al.*, 1988). The nature of these trials can be illustrated by the following example. Telch & Telch (1986) compared the effect of coping skills training with that of supportive psychotherapy in 41 patients with various cancers. These patients were selected for the trial because a clinical interview showed evidence of high psychosocial distress. They were randomly allocated to (a) a coping skills group, (b) supportive group psychotherapy and (c) a no-treatment control group. Coping skills training comprised six sessions during which patients were taught relaxation and stress management, assertive communication, cognitive restructuring and problem solving, management of feelings and planning of pleasant activities. Supportive group therapy also comprised six sessions which were non-directive and encouraged mutual sharing of feelings and concerns. Outcome was assessed at the end of the therapy. Patients who received coping skills training showed significant improvement (compared with pre-therapy scores) in anxiety and depression, in coping with medical procedures, and in satisfaction related to work, social activities, physical appearance and sexual intimacy. By comparison, patients receiving supportive psychotherapy showed little improvement and untreated patients showed significant deterioration in psychological adjustment. No follow-up results were obtained.

What, then, is the current state of knowledge in this area? The findings of a detailed review of the cited randomised controlled trials (Moorey & Greer, 1989) can be summarised as follows. First, there is general agreement that psychological therapy should be confined to those patients who show evidence, on psychological screening, of high levels of emotional distress. Second, psychological therapy is acceptable to the majority of such patients and feasible in an oncology setting. Third, five of the cited studies reported statistically significant improvement in psychiatric symptoms and/or social adjustment, but two studies failed to show any improvement. Fourth, patients with advanced as well as early cancer can benefit measurably from psychological therapy. Finally, all authors agree that psychological therapy for cancer patients should be focused on current problems. Beyond that, there is insufficient evidence to judge the relative efficacy of different kinds of therapy.

To the broad question, 'can psychological therapy improve the quality of life of patients with cancer?', the best current answer is a qualified 'yes'. The dearth of studies which meet stringent scientific requirements precludes any definitive conclusions, but there is increasing evidence to support the efficacy of psychological therapy. Methodologically rigorous research is now required to determine which specific, definable psychotherapeutic procedures can be shown to produce specified, measurable improvement in psychosocial adjustment and to identify those patients who are most likely to benefit. The methodological problems involved in trials of psychological therapy are, indeed, formidable but not insurmountable. Success will depend on the willingness and ability of oncologists and liaison psychiatrists to break down existing territorial boundaries and to collaborate closely. The laudable aim of such studies is to lay the foundation for a truly comprehensive medical service for cancer patients – in other words, a service which treats the person as well as the disease. It is encouraging to note that this is beginning to happen in some leading cancer hospitals in Britain.

### References

CAIN, E.N., KOHORN, E.I., QUINLAN, D.M., LATIMER, K. & SCHWARTZ, P.E. (1986). Psychosocial benefits of a cancer support group. *Arch. Phys. Med. Rehab.*, **61**, 128.

COURSEY, K., DAWSON, J.J. & LUCE, J.K. (1975). Comparative anxiety levels of cancer patients and family members. *Proc. Am. Assoc. Cancer Res.*, **16**, 246.

CUNNINGHAM, A.J. (1988). From neglect to support in coping: the evolution of psychosocial intervention for cancer patients. In *Stress and Breast Cancer*, Cooper, C.L. (ed) p. 135. Wiley: Chichester.

DEROGATIS, L.R., MORROW, G.R., FETTING, J. & 5 others (1983). The prevalence of psychiatric disorders among cancer patients. *JAMA*, **249**, 751.

FARASH, J.L. (1979). Effect of counseling on resolution of loss and body image following a mastectomy. *Diss. Abstr. Int.*, **39**, 4027B.

FOBAIR, P., HOPPE, R.T., BLOOM, J., COX, R., VARGHESE, A. & SPIEGEL, D. (1986). Psychosocial problems among survivors of Hodgkin's disease. *J. Clin. Oncol.*, **4**, 805.

LICHTMAN, R.R. & TAYLOR, S.E. 1986). Close relationships and the female cancer patient. In *Women with Cancer*, Anderson, B.L. (ed) p. 233. Springer: New York.

LINN, M.W., LINN, B.S. & HARRIS, R. (1982). Effects of counseling for late stage cancer patients. *Cancer*, **49**, 1048.

MAGUIRE, P., TAIT, A. BROOKE, M., THOMAS, C. & SELLWOOD, R. (1980). Effect of counselling on the psychiatric morbidity associated with mastectomy. *Br. Med. J.*, **281**, 1454.

MOOREY, S. & GREER, S. (1989). Can psychotherapy improve patients' quality of life? In *Psychological Therapy for Cancer Patients: a New Approach*. Heinemann: London.

MORRIS, T., GREER, H.S. & WHITE, P. (1977). Psychological and social adjustment to mastectomy: a two year follow-up study. *Cancer*, **40**, 2381.

SELBY, P. & ROBERTSON, B. (1987). Measurements of quality of life in patients with cancer. *Cancer Surv.*, **6**, 521.

SPIEGEL, D., BLOOM, J.R. & YALOM, I. (1981). Group support for patients with metastatic cancer. *Arch. Gen. Psychiat.*, **38**, 527.

SUGARBAKER, P. H., BAROFSKY, I., ROSENBERG, S.A. & GIANOLA, P.J. (1982). Quality of life assessment of patients in extremity sarcoma clinical trials. *Surgery*, **91**, 17.

TELCH, C.F. & TELCH, M.J. (1986). Group coping skills instruction and supportive group therapy for cancer patients: a comparison of strategies. *J. Consult. Clin. Psychol.*, **54**, 802.

WATSON, M., DENTON, S., BAUM, M. & GREER, S. (1988). Counselling breast cancer patients: a specialist nurse service. *Counsel. Psychol. Q.*, **1**, 25.